# Draft Genome Sequence of *Idiomarina* sp. Strain 5.13, a Highly Stress-Resistant Bacterium Isolated from the Southwest Indian Ridge

**DOI:** 10.1128/genomeA.01747-16

**Published:** 2017-03-09

**Authors:** Rupesh Kumar Sinha, K. P. Krishnan, P. John Kurian

**Affiliations:** National Centre for Antarctic and Ocean Research, Head land Sada, Vasco-da-Gama, Goa, India

## Abstract

*Idiomarina* sp. strain 5.13, able to produce biopolymer and exopolysaccharide, was isolated from a sediment sample collected from the Southwest Indian Ridge, Indian Ocean. Analysis of its draft genome sequence provides insights into its remarkable stress tolerance and offers the genetic basis for harnessing the biotechnological potential of this strain.

## GENOME ANNOUNCEMENT

*Idiomarina* sp. strain 5.13 was isolated from a sediment sample collected from the Southwest Indian Ridge (26°56.49′S, 67°19.477′E), Indian Ocean, and was found to produce biopolymer and exopolysaccharide (R. K. Sinha, K. P. Krishnan, and P. J. Kurian, unpublished data). Here, we report the draft genome sequence and annotation of strain 5.13. The genomic sequence was determined using the Illumina reversible terminator technology. The total sequence of 1,757 Mb with 6,955,430 mate-pair and 9,121,892 paired-end reads was generated from the HiSeq 2500 platform. During preprocessing, adapter sequences were removed using Cutadapt version 1.9 (1). All low-quality (*Q* < 20) data were filtered out using Sickle version 1.33. Contigs lesser than 200 bp and duplicate reads were discarded using Fastuniq (2). KmerGenie (3) was used to predict the optimal *k* value and assembly size, which was found to be 31 and 7,352,014 bp, respectively. *De novo* assembly was performed with Velvet version 1.2.10 (4) using default options, except the *k* value, which resulted in 87 contigs.

*Idiomarina* sp. strain 5.13 has genome length of ~4.5 Mb with a G+C content of 46.1% and no plasmids, as deduced with PlasmidFinder (5). The assembly was annotated using Prokka (6), which predicts coding DNA sequence (CDS) using Prodigal (7). A total of 4,043 CDSs were predicted. The predicted CDSs were annotated using the UniProt database (http://www.uniprot.org/) of the BLAST program, followed by comparison with the HMM profile database and Pfam database (8) using the HMMER3 program (9). Matches with an *E* value of ≤10^-6^ were retained for further annotation. A total of 1,049 hits were labeled as hypothetical proteins by the Prokka program. tRNA and transfer-messenger RNA (tmRNA) genes predicted from the contigs using Prokka, which uses Aragorn (10), found 52 tRNA genes and one tmRNA gene. The genome sequence of strain 5.13 showed the presence of phage remnant-like transcription regulator AlpA and phage packaging genetic machinery (*gpAZ nu1*). The presence of 66 transposases and phage integrase family protein scattered throughout the genome supported the likelihood of active genetic rearrangement of genome of strain 5.13. The bacterium is equipped with resistance genes against heavy metals, such as mercury (*merACPRT*), cobalt, zinc, and cadmium (*czcAB*, *zur1*, *zur2*, *zntB*, *zupT*, *zitB,* and *zip* family), arsenic (*arsC*), copper (*copABCD*), nickel (*nikA*), and manganese (*mntHP*), and possesses a cation efflux genetic system (*cusAB* and *cutA*). It contained 69 genes for chemotaxis and flagellar motility. *Idiomarina* sp. strain 5.13 can accumulate polyhydroxybutyrate in intracellular storage granules (R. K. Sinha, K. P. Krishnan, and P. J. Kurian, unpublished data) conferred by the presence of *phbC* and a phasin protein family. Genetic components for exopolysaccharide production and biofilm formation and dispersion (*exoD*, *ycgF1*, *ycgF2*, *bigR*, and *bdIA123*) were also present. Genes that could allow strain 5.13 to cope with oxidative stress and cold shock (*phoH*, stringent starvation protein family, *cspAGD*) were observed, along with eight chaperone-encoding genes.

The overall genome analysis revealed a high potential of strain 5.13 in bioremediation and biotechnological application and provides insight into its adaptability to extreme environmental conditions.

### Accession number(s).

This whole-genome shotgun project has been deposited at DDBJ/ENA/GenBank under the accession no. MSEI00000000. The version described in this paper is version MSEI01000000.
